# Phase III evaluation of the insecticidal efficacy and durability of a deltamethrin-treated polypropylene long-lasting net LifeNet®, in comparison with long-lasting nets made from polyester and polyethylene: study protocol

**DOI:** 10.1186/s13690-016-0168-2

**Published:** 2016-12-30

**Authors:** Patrick Tungu, Louisa A. Messenger, Matthew J. Kirby, Wema Sudi, William Kisinza, Mark Rowland

**Affiliations:** 1 National Institute for Medical Research, Amani Medical Research Centre, Muheza, Tanzania; 2 Department of Disease Control, London School of Hygiene and Tropical Medicine, London, UK

**Keywords:** Long-lasting insecticidal nets, Malaria control, Phase III, Net durability, Attrition, Bioefficacy, LifeNet®, WHOPES, Deltamethrin

## Abstract

**Background:**

Universal coverage of long-lasting insecticidal nets (LNs) made from polyester or polyethylene fibres has been adopted as the standard of care to control malaria among at-risk populations. To obtain a WHO recommendation, LNs must undergo prospective monitoring of insecticidal efficacy against mosquito vectors over 3 years of household use. The retention of bioefficacy and physical durability of a LN is influenced by net usage practices, textile polymer material and insecticide treatment technology. Fabric durability is the critical factor which determines the interval required between LN replacement campaigns. To investigate factors known to affect LN durability and bioefficacy, we describe a three-arm WHO Pesticide Evaluation Scheme (WHOPES) Phase III evaluation of a LN made uniquely from polypropylene (LifeNet®, Bayer CropScience) compared to standard LNs made from polyester and polyethylene, all treated with deltamethrin, over 3 years of use.

**Methods:**

This is a prospective three-arm household randomized, equivalence trial of LNs in Tanzania, with nets as the unit of observation. Equal numbers of houses will be randomized to receive deltamethrin-treated polypropylene, polyester or polyethylene LNs; all sleeping spaces in a given household will be provided with one type of net. Bioefficacy (insecticidal activity against mosquitoes), insecticide content of net fibres, and fabric integrity (number, location and size of holes) will be measured every 6 months, using WHO cone or tunnel bioassays, chemical analysis and calculation of hole index, respectively. A cohort of LNs will be surveyed annually to assess survivorship (median LN survival time) and cumulative loss of fabric integrity. Field durability outcomes will be compared with laboratory strength tests.

**Discussion:**

This is the first trial to compare the relative durability of three LNs each made from a different textile polymer, treated with the same insecticide, in the same community side-by-side over 3 years of use. Trial findings will 1) guide global health organizations on procurement policy and the type of textile polymer which maximizes the interval between LN replacement campaigns, and 2) stimulate manufacturers to improve product performance and development of longer lasting polymers. A full WHO recommendation may be granted to LifeNet® upon successful Phase III completion.

## Background

Long-lasting insecticidal nets (LNs) are one of the key measures recommended by the World Health Organization (WHO) for prevention and control of malaria [1]. While considerable progress has been made by many National Malaria Control Programs (NMCPs) and international donor agencies to facilitate free or subsidized universal coverage campaigns (UCCs) for at-risk populations [2], of increasing concern is how to prolong effective life and field durability of LNs in order to extend the interval between UCCs and improve cost-effectiveness [3].

In recent years, the WHO has developed guidelines to assess the insecticidal efficacy of LNs, net durability and survivorship under operational conditions [4–6]. New LNs are granted a WHO Pesticide Evaluation Scheme (WHOPES) time-limited interim recommendation for use following successful evaluations in laboratory conditions (Phase I) and in small-scale field trials in experimental huts (Phase II), during which a product must retain insecticidal activity for at least 20 standardized washes with respect to vector knock-down, mortality and blood-feeding inhibition [7, 8]. To receive a full WHO recommendation, LNs are required to undergo prospective insecticidal efficacy and net durability monitoring over 3 years of household use (Phase III) [7, 8]. LN durability is measured as a function of net survivorship, fabric integrity and insecticidal activity [4], which are all influenced by frequency of use, net care and repair, washing and maintenance practices, duration of transmission season [9–11], as well as the textile’s physical features, such as fibre material, knitting or weaving pattern, insecticide type and concentration, and fibre impregnation technology [12]. Average effective net life is often assumed to be 3 years [4] but can vary substantially between products [13, 14], endemic regions [15–19], and conditions of use [20].

The potential market for LNs in Tanzania, as in all African malaria endemic countries, is large and significant [1, 21]. Since 2004, the NMCP in Tanzania, supported by the Global Fund to Fight AIDS, Tuberculosis and Malaria (GFATM), has scaled up the distribution of LNs through a range of public and private sector mechanisms [22], most notably the Under Five Coverage Campaign launched in 2008 [23] and the UCCs of 2010-11 and 2015-16 to provide full coverage in all households at risk of malaria [24].

While a WHOPES Phase III recommendation is based primarily on insecticidal efficacy over time, the physical durability of the fabric is the main factor determining the lifespan of a typical LN. Durability is influenced by fibre denier, the bursting strength of the net, the knitting pattern and the polymer material [12]. All brands of LN in regular use are made from either polyester (43%) or polyethylene (57%) [25]. LifeNet® (Bayer CropScience, Germany) is a new deltamethrin-impregnated long-lasting net (LN), uniquely made from polypropylene knitted fibres, which received interim WHOPES recommendation in 2011 following successful Phase I and Phase II trials [26, 27]. According to the manufacturer the main advantage over standard polyester and polyethylene LNs is its improved durability for household use [26]. To investigate factors known to affect LN durability, here we describe the study design and methodology of a large scale community-based Phase III evaluation of LifeNet® LN compared to two WHOPES recommended LNs made from polyester and polyethylene respectively, all treated with the same insecticide (deltamethrin), over 3 years of field use in North-East Tanzania.

### Study objectives

To undertake a large scale community-based Phase III evaluation comparing the insecticidal efficacy and durability of polypropylene LifeNet® LN against a deltamethrin-treated polyester LN with full WHOPES approval (PermaNet® 2.0) and a deltamethrin-impregnated polyethylene LN over 3 years of continuous use in a malaria endemic setting of North-East Tanzania.

Specific objectives

- To assess the insecticidal efficacy of a deltamethrin-treated LN made of polypropylene (LifeNet®) compared with deltamethrin-treated LNs made of polyester and polyethylene over 3 years of household use.
- To determine the durability (survivorship and fabric integrity) of a deltamethrin-treated LN made of polypropylene (LifeNet®) compared with deltamethrin-treated LNs made of polyester and polyethylene over 3 years of household use.
- To assess washing habits and frequency of LN washing by householders over 3 years of household use.
- To document and compare community perceptions of all three LNs by participants over 3 years of household use.

All objectives will be investigated following standardized WHO guidelines for laboratory and field testing of LNs [28] and durability monitoring under operational conditions [4].

## Methods/Design

### Study area and participant recruitment

**Fig. 1:**
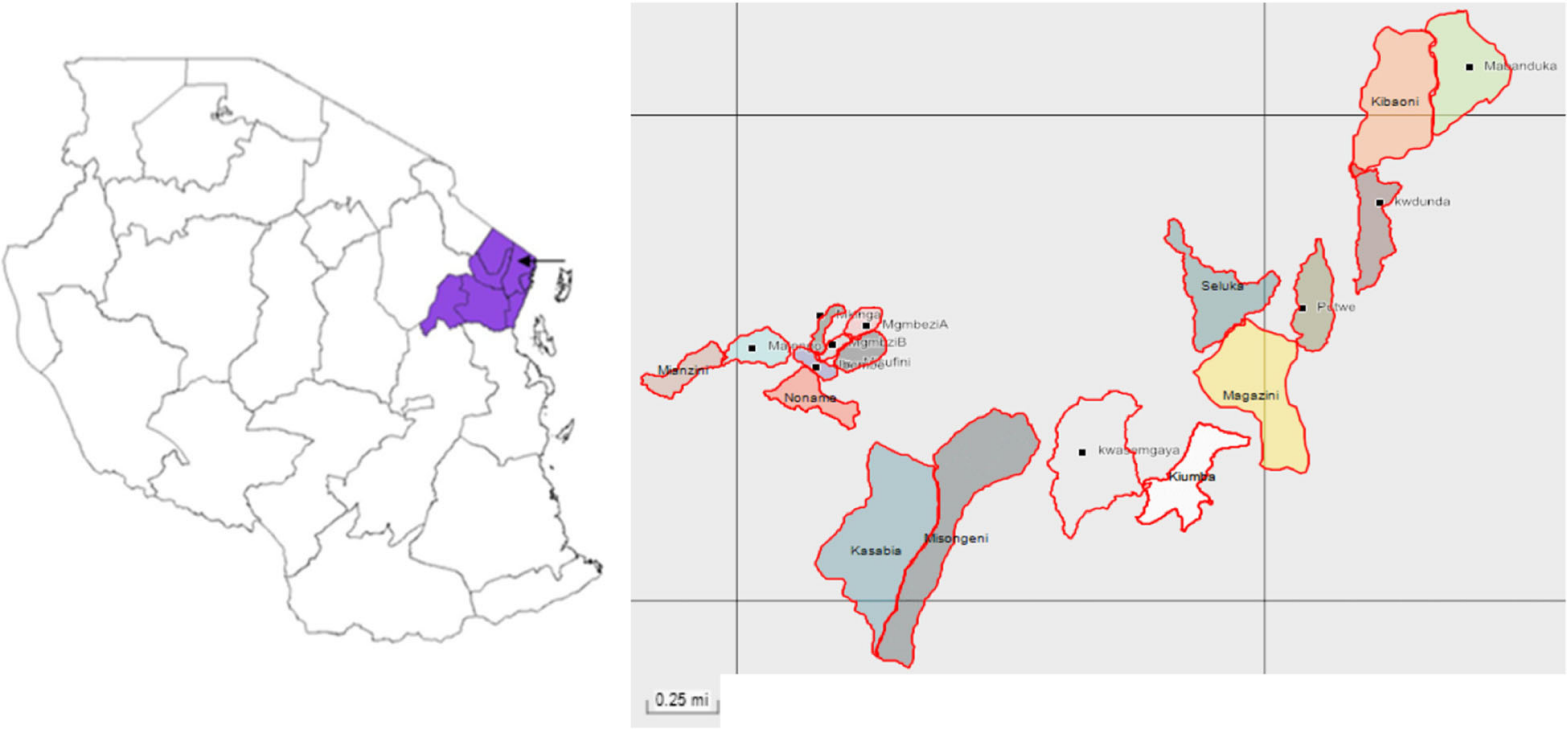
Map showing 18 study hamlets identified in Muheza District (*arrow*), Tanga region (*shaded*), North-East Tanzania

The trial will be conducted in four villages (Magila, Kibaoni, Ubembe and Misongeni) situated in Muheza District, Tanga region of North-East Tanzania (5^o^ S, 39^o^ E) (Fig. 1). The district encompasses an area of approximately 4922 km^2^, ranging from a coastal plain at sea level to the Usambara mountains at 1500 m. The climate is tropical with dense forest covering the mountain region. Muheza District has four administrative divisions comprising 33 wards with 135 villages, mainly inhabited by subsistence farmers. The population of Muheza District was 204,461 residents in 2012 with an annual growth rate of 2.2% [29]. The average population size of a typical village is approximately 1700 inhabitants among 450 households.

Muheza District is highly endemic for malaria with transmission occurring throughout the year with two seasonal peaks during and after the long rainy season from July to August and the short rainy season from December to January [30]. During the rainy seasons *Anopheles gambiae* sensu stricto and *An. funestus* are the most abundant malaria vectors [31]. During the dry season *An. funestus* remains fairly common but *An. gambiae s.s.* numbers decline [31–33]. The lymphatic filariasis vector and nuisance mosquito *Culex quinquefasciatus* is abundant from December to June. Historically, Muheza District has not been subjected to IRS but did receive LNs during nationwide UCC distributions [23, 24].

Villages or groups of larger hamlets will be selected in collaboration with the District Assembly and District Medical Officers (DMOs) on the basis of population size, number of households, willingness to participate, road accessibility and proximity to the National Institute for Medical Research (NIMR) laboratory and insectary facilities of Amani Medical Research Centre and with no other ongoing vector control interventions at time of recruitment.

### Study design, randomization, blinding and bias

This will be a prospective cluster randomized controlled, equivalence trial with nets as the unit of observation. Following household enumeration, global positioning system (GPS) coordinates will be recorded and baseline socio-demographic and economic data, including age, sex, occupation, education, household wealth, sleeping habits and number of spaces, ownership and use of other mosquito nets and net preferences (size, colour, shape and washing habits) will be collected using a standardized questionnaire.

Once baseline chemical analysis of LNs is completed and confirmed to be within WHO specifications, the nets will be distributed. The monitoring period will be 3 years, beginning August 2014 (Table 1). At study onset, equal numbers of households from eighteen hamlets will be randomized to the three study arms. All participating households in a specific hamlet will be provided with the same type of LN to achieve universal coverage, assuming an average of 4.0 sleepers and 2.1 sleeping places per household. To ensure all members in a participating household are using the same LN brand, any existing nets will be removed and replaced by study LNs. All LNs will be issued free of charge to recipients. Net users will be encouraged to retain the LNs they are given and not to sell or exchange them with other study participants. If for any reason an owner stops using a net, they are requested to volunteer the reason and to store the net for later inspection by investigators.

**Table 1:** Timetable of study activities

Activity	Pre-Intervention	Post Intervention monitoring period (months)						
		0	6	12	18	24	30	36
Community selection, sensitization and consent	X							
Baseline census and household survey	X							
LLIN distribution	X							
Cross-sectional surveys: bioefficacy assays	X	X	X	X	X	X	X	X
Cross-sectional surveys: chemical assays	X			X		X		X
Cross-sectional surveys: fabric integrity		X	X	X	X	X	X	X
Cross-sectional surveys: fabric strength		X	X	X	X	X	X	X
Cross-sectional surveys: community practices		X	X	X	X	X	X	X
Cohort surveys: household survivorship and fabric integrity		X	X	X		X		X
Insecticide resistance monitoring	X			X		X		X
Adverse events monitoring		X						

**Fig. 2:**
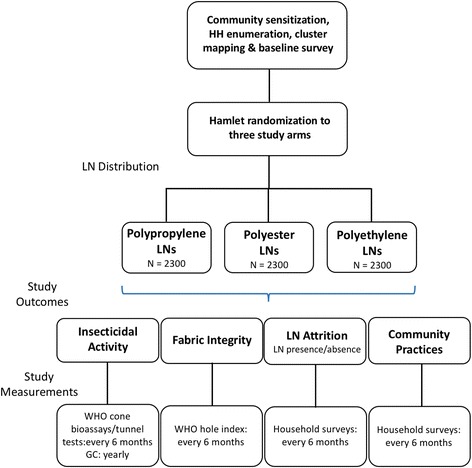
Schematic of study design

To assess bioefficacy (insecticidal activity measured by mosquito knock-down and mortality), deltamethrin content (g/kg and mg/m^2^), fabric integrity (location and size of holes) and fabric strength, a series of cross-sectional surveys and destructive sampling of LNs will be undertaken on randomly selected nets every 6 months for WHO cone bioassays, yearly for insecticide analysis using gas chromatography (GC), and physical integrity (hole index) every 6 months up to 18 months and then annually. A longitudinal cohort of LNs will be followed every 6 months to measure survivorship (net attrition rate) and loss of fabric integrity. Community attitudes and practices of LN use will be assessed every 6 months using the standard WHO LN durability monitoring questionnaire. Impact of the interventions on insecticide susceptibility of major malaria vectors will be evaluated each year in study villages and neighboring control areas. Study participants will also be monitored for possible adverse events during the first month post LN distribution using a structured questionnaire. A schematic representation of the trial is shown in Fig. 2.

Observer bias will be reduced where feasible. However, there are limits because the material of each type of net is distinct to touch. Manufacturers’ labels will be removed before distribution and nets allocated using a unique nine-digit numerical identification (ID) code, composed of village (3), hamlet (2), household (3) and net (1) numbers, stenciled directly onto the LN using permanent dye. Separate IDs will be issued to those LNs assigned to the cohort study. Technicians conducting the bioefficacy, fabric integrity assays and survivorship surveys will be blinded to the brand of LN.

During destructive sampling, it is important to replace LNs with nets of the same brand to measure the true rate of loss of fabric integrity. For example, households using a specific LN may prefer another type of net given freedom of choice and use that in preference to any of the former nets still present in the house. This could result in nets of the former type developing holes less rapidly and appearing artificially more ‘durable’, thereby introducing an avoidable bias.

Fabric integrity will be assessed using two methods, by following a longitudinal cohort and cross-sectional surveys. This should detect any potential user bias resulting from the ‘Hawthorne effect’, the possibility that regular visits render participants more inclined to retain or care for their nets and less likely to dispose of them when damaged because they are being observed.

### LN products and distribution

LifeNet® LN is a net with deltamethrin-incorporated into 100 denier polypropylene filaments at 8.5 g AI/kg or 340 mg/m^2^ (Bayer CropScience, Germany) [26]. It has undergone a WHO generic risk assessment model and does not pose undue hazards to the user when used as instructed [34]. LifeNet® LN has attained a WHO interim recommendation following successful Phase II experimental hut trials in 2011 [26] and will be granted a full WHO recommendation following successful completion of a Phase III evaluation.

Under WHOPES guidelines, LifeNet® LN efficacy and durability will be compared to a LN which has already completed Phase III evaluations successfully. PermaNet® 2.0 LN (Vestergaard Franden, Switzerland) is a multifilament polyester net treated with deltamethrin to a target concentration of 55 mg/m^2^, bound in a wash-resistant resin coating that received full recommendation in 2008 and is widely used by NMCPs [35]. Because LifeNet® LN introduces a novel polymer to the range of textile materials currently favoured for nets it is important to test the polypropylene LN against net products representative of different material types. In addition to a polyester LN typified by PermaNet® 2.0 LN, LifeNet® LN will be compared with a net brand submitted to WHOPES, made from polyethylene monofilaments incorporated with deltamethrin to a target concentration of 63 mg/m^2^ (Table 2). This study will provide a unique opportunity to evaluate the relative durability and residual efficacy of three LNs each made from different textiles, containing the same insecticide (deltamethrin), following two different treatment methods (incorporation and coating).

**Table 2:** Characteristics of three LN brands distributed in the study

LLIN Brand	Manufacturer	Fabric	Deltamethrin Concentration	A.I. Application	Denier	Av. Mesh Size Per cm^2^	Bursting Strength (kPa)	Colour	Size (cm)	WHOPES Recommendation	References
LifeNet®	Bayer CropScience, Germany	Polypropylene	340 mg/m^2^	Incorporated into fibres	100	21–29	450	White	W190 × L180 × H 150	Interim (2011)	[26]
PermaNet® 2.0	Vestergaard Frandsen, Switzerland	Polyester	55 mg/m^2^	Resin coating	100	24	250–350	Blue	W190 × L180 × H 150	Full (2008)	[35]
Polyethylene LN	Bestnet A/C, Denmark	Polyethylene	63 mg/m^2^	Incorporated into fibres	110	21	540	White	W190 × L180 × H 150	Withdrawn (2014)	[46]

LNs will be distributed using a voucher scheme; study team members will visit participating households door-to-door and distribute coupons which specify the type and number of nets to be received from a focal point in each hamlet. At the time of LN distribution, householders will be asked to begin using their nets immediately, to store and not use any existing nets and reminded about appropriate use and maintenance of their nets; storage of existing nets will be verified by house visits 1 month post LN distribution. Study teams will also offer assistance to erect nets over sleeping areas. Participants will be advised to report any adverse effects, including headache, numbness, itching, sneezing, nasal discharge, discharge from eyes, nausea and unpleasant smells, and to seek medical care from the nearest health facility if the symptoms persist or if they observe any signs of malaria or other vector-borne diseases. Study teams will offer assistance and reiterate this information every time a LN is withdrawn for laboratory analysis and replaced throughout the monitoring period.

### Cross-sectional surveys: bioefficacy

To determine the insecticidal efficacy of each intervention, at the beginning of the study (t0) and every 6 months thereafter (t6, 12, 18, 24, 30), 30 LNs of each brand will be randomly selected from the net master list and assessed using standard WHO cone bioassays [8]; 50 LNs of each brand will be drawn randomly for the survey at 36 months (t36). LNs will be removed from participating households and replaced with new nets of the same type.

**Fig. 3:**
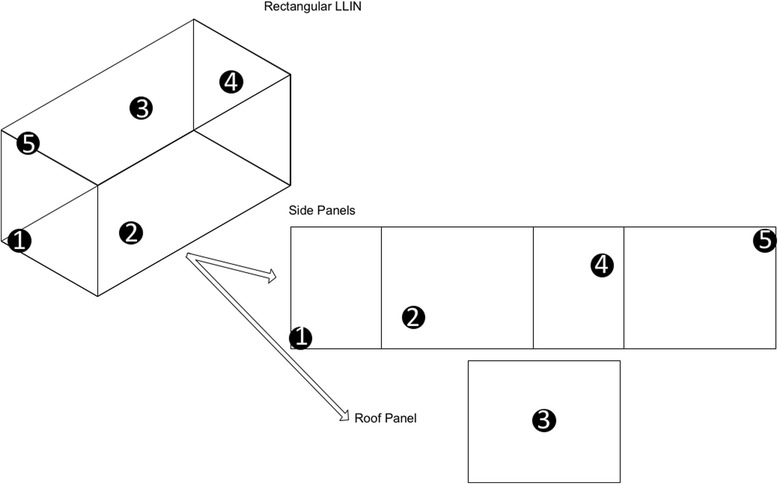
Sampling scheme for LNs

From each LN, five samples (25 × 25 cm) will be cut at positions 1 to 5 (Fig. 3). All bioassays will be performed using 2–5 day old non-blood fed, laboratory-reared susceptible *An. gambiae s.s.* Kisumu strain. Twenty mosquitoes will be exposed to each netting sample, in four standard WHO cones fixed with a plastic manifold, for 3 min and then held for 24 h in paper cups with cotton wool soaked with 10% sugar solution; knock-down will be recorded after 60 min and mortality after 24 h. Mosquitoes exposed to an untreated net will be used as a negative control in each round of assays. All bioassays will be performed at 25 ± 2 °C and 75 ± 10% relative humidity.

Bioassay data for position 1 will be analysed separately from the others (2–5) considering this part of the LN is subjected to excessive abrasion during routine use; this portion of net is frequently manipulated while tucking the LN under the bed/mattress. Likewise, no chemical analysis will be conducted on position 1 samples. If there are significant variations between bioassay results, mean results of positions 2–5 will only be used.

If LNs do not meet the efficacy criteria of ≥95% knock-down rate after 60 min or mortality of ≥80% after 24 h in the cone bioassays, they will be subjected to a tunnel test using a guinea pig as bait to determine whether they meet the efficacy criteria of ≥80% mortality or ≥90% blood feeding inhibition [28]. The LN piece will be attached to a disposable cardboard frame, placed at one third of the length of a glass tunnel measuring 25 cm^2^ × 60 cm, exposing 400 cm^2^ (20 × 20 cm) of surface netting. Nine holes, each 1 cm in diameter (one in the centre of the square and the other eight equidistant at 5 cm from the border) will be made in the netting to allow for passage of mosquitoes; a netting-covered cage is attached at each end of the tunnel to introduce mosquitoes. In the shorter section of the tunnel, a guinea pig will be restrained and one hundred 5–8 day old non-blood fed *An. gambiae s.s.* Kisumu mosquitoes will be introduced from a cage positioned at the longer end of the tunnel. The experiment will begin at 18:00 and end at 09:00 the following morning, after which mosquitoes will be scored according to whether they passed through the netting, whether they successfully blood fed and whether they survived the exposure period. The test will be replicated twice with 50 mosquitoes per test using the piece of netting from the failed LN that gave a cone mortality result closest to the average obtained for positions 2–5. All tunnel tests will be performed at 25 ± 2 °C and 75 ± 10% relative humidity under subdued light. A tunnel with untreated netting will be used as a negative control. Because blood-feeding rate in the negative control influences contact and mortality with the pyrethroid-treated samples, a minimum blood-feeding rate of 50% in the controls is required in the paired treatment-control tunnel tests.

### Cross-sectional surveys: chemical analysis

Samples of the three types of LNs will be subjected to chemical residue analysis at the WHO Collaborating Centre for quality control of pesticides (Gembloux, Belgium) at the beginning of the trial (t0) to ensure that the target dose of deltamethrin is correct and at yearly intervals (t12, 24 and 36) to facilitate interpretation of bioassay data. From the 30 LNs sampled for bioassays at t12 and t24 months and the 50 LNs sampled at t36 months, additional 30 × 30 cm sections will be cut from positions 2–5 (Fig. 3) to estimate total deltamethrin content in the net (expressed in both g/kg and mg/m^2^). Samples will be rolled up and wrapped in labelled, aluminium foil and stored at 4 °C, prior to extraction of deltamethrin and analysis by GC according to the Collaborative International Pesticides Analytical Council (CIPAC) protocol [36].

### Cross-sectional surveys: fabric integrity

At t6, 12 and 18 months post LN distribution, the 30 LNs destructively sampled for bioassays will be scored for physical integrity by draping the nets over a frame and counting the number and types (due to burning, tearing, split seams or animal damage) of holes of different sizes according to location on the net (top, upper side, lower side). Holes greater than 0.5 cm will be classified into the following categories: smaller than a thumb (0.5–2 cm); larger than a thumb but smaller than a fist (2–10 cm); larger than a fist but smaller than a head (10–25 cm) and larger than a head (>25 cm). At t24 and 36, physical integrity of the nets will be evaluated for a randomly selected sample of 100 LNs of each brand (including the 30 destructively sampled for bioassays). Net integrity (hole index, hole area and circumference) of each net type will be calculated as per WHO guidelines [28] (Table 3). In addition, any evidence of repairs and types of modification will be recorded.

**Table 3:** LN hole size index

Hole Size Categories (cm)	Hole Diameter^a^ (*d;* cm)	Hole radius (*r* = *d/*2)	Hole Area^b^ (π*r ^2^)	Hole Index^c^
0.5–2.0	1.25	0.625	1.23	1
2–10	6	3	28.28	23
10–25	17.5	8.75	240.56	196
>25	30	15	706.95	576

^a^Weights for each size category were estimated assuming that the average hole diameter was the midpoint in each category, except for the largest hole where the average diameter was assumed to be 30 cm

^b^The approximate area of an average sized hole from each category was estimated by assuming each hole was approximately circular

^c^Final weights for each size category were then estimated by dividing the area encompassed by a hole in the smallest size category (i.e. 1.23)

### Cross-sectional surveys: fabric strength

At t6, 12, 18, 24, 30 and 36 months post LN distribution, when 30 LNs of each brand are destructively sampled for bioassays, the remaining part of each net will be packaged and sent to CITEVE, Portugal to conduct a series of tests to determine the strength of netting fabric and resistance-to-damage as recommended by the WHO consultation on the determination of fabric strength of LNs [37]. These tests will include bursting strength (pneumatic), wounded bursting strength, hook tensile strength, snag strength, abrasion resistance and hole propagation tests. These measures of snagging, tearing, holing and hole enlargement will be correlated with measures of net durability and hole index during 3 years of household use.

### Cross-sectional surveys: community practices

At t6, 12, 18, 24, 30 and 36 months post LN distribution, one adult participant from each of 90 of the households selected for fabric integrity surveys (30 per study arm), will be interviewed to assess net utilization practices (including early morning observations), method and number of washes and type of soap used, as per the standard WHO LN durability monitoring questionnaire [28].

### Cohort study: household survivorship and fabric integrity

To measure LN household survivorship and attrition, a longitudinal cohort of 250 LNs from 125 randomly selected households (two nets per household) will be followed at t6, 12, 18, 24, 30 and 36 months post LN distribution. At each time point, 150 of the LNs (the higher of the 2 ID codes per house) will be examined to measure the loss of fabric integrity over time (hole index, as above) and net hanging (net usage), and attrition (physical presence/absence of the nets) of all household LNs will be assessed. When a LN is missing from a house, the participants will be questioned to determine the reason for its loss or absence, which will be categorised as: loss by giving away the LN to others, sold, stolen, mislaid, using for alternative purposes or discarded due to loss of integrity (wear and tear); the latter is most important for assessing the relative durability of each type of net. LN owners will be requested to retain and store the net if they stop using it and to allow its inspection during follow up surveys. At the end of the 3 year trial period, study nets will be replaced on their production regardless of condition. Study LNs in the house that have never been used will be recorded but excluded from the analysis.

In contrast to the cross-sectional surveys, where only surviving LNs will be examined, the cohort study will allow gradual deterioration of individual LNs to be monitored, and the point at which the net is discarded or no longer used, determined. Households participating in the cohort study will not be sampled during cross-sectional surveys.

### Adverse events monitoring

The frequency of adverse events (AEs) will be monitored among 100 randomly selected households from each of the three study arms using a structured questionnaire at 1 month following LN distribution. During these house visits, the study team will also assess LN usage rates and ensure LNs are being hung properly.

### Insecticide resistance monitoring

Susceptibility of *An. gambiae s.l.* and *An. funestus* to deltamethrin will be measured at baseline and annually throughout the monitoring period both in study villages and neighbouring areas without study LNs, using WHO susceptibility tests [38].

### Project oversight, safety considerations and handling withdrawals/drop-outs

There are no apparent risks to the safety of study participants and communities. All three products will be used in compliance with WHO recommended guidelines [26, 35, 39]. Project progress and any issues arising will be subject to annual review under the WHO Continuing Ethical Review process. While each brand of LN is expected to provide protection from malaria, participants will be informed that they should visit the nearest health facility or district hospital for diagnosis, upon experiencing fever or any other malaria symptoms. Additionally, the Principal Investigator (PI) will inform the DMO about possible reporting of AEs and to provide medical care, as necessary.

Participants and households are free to withdraw from the study at any time with impunity and will be allowed to retain their LNs. Net owners who have withdrawn consent, or moved out of the study area and have taken their LNs with them, will be replaced by another net user from the same or neighboring household within the hamlet.

### Sample size rationale

Sample size calculations are based on detecting differences in LN insecticide efficacy and physical durability (attrition rate and fabric integrity) between the three comparison nets over a 3 year period, while allowing for destructive sampling and replacement of LNs for biological and chemical efficacy assays and an expected attrition rate of 20% per year for each product.

#### Cross-sectional surveys: biological and chemical efficacy and fabric integrity

During cross-sectional surveys, one LN will be sampled from 30 households per study arm per survey at t0, 6, 12, 18, 24 and 30 months and from 50 households at 36 months, thus requiring a minimum of 320 households per intervention arm, factoring in an attrition rate of 20% per year (total of 960 households). Allowing for distribution of 3.2 nets per house (universal coverage of participating households) plus replacements at the time of net withdrawal for bioassays/chemical assays and possible attrition in use, the total number of LNs required is 1634 per study arm. To assess fabric integrity, a total of 50 LNs will be destructively sampled per arm per survey; 30 of these will be the same nets collected for bioassays.

#### Cohort surveys: household survivorship and fabric integrity

During the longitudinal cohort study, 250 LNs from 125 households per study arm will be monitored after 6 months and then followed at annual intervals to evaluate net survivorship and attrition and loss of fabric integrity over 3 years. All 250 LNs will be assessed for survivorship and 150 (approximately one per household) will be examined for loss of fabric integrity. Assuming an attrition rate of 20% per year, 200 households will participate in the cohort study (an additional 640 LNs), allowing a 12% point difference in LN attrition rate to be detected.

A total of 2300 nets per study arm will be required for both cross-sectional and cohort surveillance.

### Data handling and record keeping

All demographic data will be recorded by Tanzanian fieldworkers using Android smartphones containing pre-programmed, pre-tested, standardized data entry forms which will be uploaded directly to an electronic server. Each LN will have a unique ID number and study subjects participating in the cross-sectional surveys will be identified by their demographic enumeration number. All forms and datasets will record participant data using these codes; no personal identifiers will be entered.

All data computers will be password protected with restricted access to only authorized study investigators and data management staff; field workers will have no access to the server. Only the PI and senior field investigators will have access to the full master LN list. A copy of the master list without household identifiers will be submitted to WHOPES. Laboratory data will be recorded on standardized forms and double-entered, firstly by field workers and then by dedicated data entry staff, and the entries combined and errors corrected to produce a single dataset. Daily data checks will be performed to identify incomplete, missing, inaccurate or inconsistent data. Datasets will be checked for consistencies by generic and study specific algorithms designed to identify sources of error. When inconsistencies arise, these will be compared to the original forms and subsequently rectified by the appropriate study investigator.

Demographic and entomological data will be kept separately from that containing personal information. Data will be stored for at least 10 years as per standard NIMR practice. The PI will maintain appropriate medical and research records in compliance with good clinical practice (GCP) and regulatory and institutional requirements. Authorized representatives of the sponsor, the ethics committee(s) or other regulatory bodies may inspect all documents and records at any time. The PI will ensure access to facilities and records, as required.

### Data analysis

All statistical analyses will be performed using STATA version 13 (StataCorp LP, College Station, TX, USA).

Bioefficacy data from the WHO cone tests will be used to calculate proportional knock-down and mortality. Bioefficacy results from the netting pieces of each LN will be pooled to determine whether the net meets the WHO efficacy criteria of ≥80% mortality or ≥95% knockdown. Bioefficacy data from the WHO tunnel tests will be used to determine whether nets that fail the cone test meet the WHO efficacy criteria of ≥80% mortality or ≥90% blood-feeding inhibition. Blood-feeding inhibition will be assessed by comparing the proportion of blood fed females (alive or dead) in treated and control tunnels. Overall mortality will be measured by pooling the immediate and delayed (24 h) mortalities of mosquitoes from the two sections of the tunnel, relative to the control. Mortality data in both WHO cone and tunnel tests will be corrected for negative control mortality using Abbott’s formula.

A candidate LN product must achieve the WHO requirement of at least 80% of sample nets meeting the bioefficacy criteria in the WHO cone bioassays or tunnel tests after 3 years of use.

Chemical analysis results will measure the mean (and standard deviation) target deltamethrin concentration in each type of LN to assist interpretation of the bioefficacy data. Insecticide content reported at each survey time will be used to estimate the average rate of insecticide loss from the original loading dose.

LN survivorship will be measured at each timepoint as the total number of each LN present in surveyed households (and used for sleeping under) divided by the total number of each LN originally distributed to households and not given away at each time point [6]. Median LN survival is the time point at which the estimate of functional LN survival reaches 50%. LN attrition is derived from the total number of LNs missing from each household (classified into categories of nets that have been destroyed or disposed for reasons other than poor fabric integrity, nets used for other purposes and nets destroyed for reasons of poor fabric integrity) divided by the total number of each LN distributed to households. LNs retained for inspection but no longer used for reasons of poor integrity will be included among the nets lost to attrition.

Fabric integrity during the field trial will be assessed using various criteria: 1) the proportion of LNs with holes (total number of each LN product with at least one hole of any size, divided by the total number of each LN product surveyed); 2) the proportional hole index (calculated by weighting each hole by size and summing for each net (Table 3) [28]; and 3) hole area and circumference. For each LN product, fabric integrity indices will be compared by analysis of variance for normally distributed data or the Kruskal-Wallis test or Poisson regression for non-parametric data.

Multivariate regression analysis will be used to identify factors that influence LN durability and community acceptability of each net product, using variables derived from household questionnaire data.

Fabric strength and resistance-to-damage tests [37] will be based on bursting strength and wounded bursting strength (EN ISO 13938-2, a measure of tear resistance), hook tensile strength (ISO 13934-2), snag strength (adapted EN 15598), abrasion resistance (ISO 12947:1998) and hole propagation tests (multi axial loading of wounded bursting strength test) of LNs collected from the field and will be correlated with LN durability indices.

## Discussion

A recent WHO consultation to review the evidence on the fabric strength of LNs concluded that the current data on the durability of LNs are inadequate, variable and poor quality, and that direct prospective trials to compare different brands of net in a variety of field settings are necessary [37]. To our knowledge the present proposed trial is the first to compare three types of LN polymer – polyester, polyethylene and polypropylene - in the same location using a prospective randomized controlled trial design.

WHOPES Phase III bioefficacy guidelines for LNs are currently based on retention of bioefficacy rather than physical durability. To obtain a WHOPES full recommendation at least 80% of the LNs surviving 3 years of household use in malaria endemic countries must retain sufficient insecticidal activity to induce 80% mosquito mortality in bioassay tests [28]. Four brands of LN have already achieved this criteria: polyester nets, PermaNet 2.0® and Interceptor® [35, 40], and polyethylene nets, Olyset® and DuraNet® [41, 42]. A further four LNs have obtained WHO full recommendations on the basis of equivalence to the aforementioned brands, and a further eight have obtained WHO interim recommendation after demonstrating bioefficacy in Phase II experimental hut trials [25]. While most brands of LN may achieve the requisite levels of bioefficacy, it is much less clear whether all types of net can physically withstand the wear and tear of 3 years of household use. LN survival will depend on the environment and household conditions of use. A LN stretched over a rustic wooden bed in a cramped traditional mud house will not last as long as a properly fitted LN draped over a bespoke net frame in a rodent free home. The duration of the mosquito season will also affect LN longevity and usage, and this will differ between the tropics and subtropics, highlands and lowlands, and coastal and plain areas [4, 15]. In practice, few brands of LN have undergone rigorous longitudinal monitoring. The first LNs to obtain WHOPES full recommendation - polyester PermaNet 2.0® LN and polyethylene Olyset® LN - were the subject of annual cross-sectional surveys of random samples of nets [35, 42] across a number of countries after 1–3 years of use rather than longitudinal monitoring of a documented cohorts of nets [28]. In these surveys it was noted that net integrity (hole index) reached a steady state after 2 years of use indicating that LNs with higher hole indexes were being discarded and not surviving to 3 years. In these countries only one brand of net had been distributed. Unless different types of LNs are tested in the same location, in parallel, in household randomized trials, it will be difficult to determine their relative durability under field conditions. Few LN brands have been evaluated against one another in this way. A recent exception was a household randomized trial in Cambodia between polyethylene Netprotect® LNs and polyester PermaNet 2.0® LNs [43]. Net survivorship exceeded 90% in the first 2 years but after 3 years decreased to ~60% for both LNs, indicating that nets of either material were unable to last much longer than 2 years. It is therefore appropriate that the proposed trial of a longer lasting polypropylene LN is assessed alongside and against polyester and polyethylene LNs over 3 years of field use.

A standard WHOPES phase III trial of a LN normally compares the candidate LN to a reference LN (positive control) made from the same material to determine insecticide bioefficacy after 3 years of field use [28]. While the proposed field trial uses the WHOPES phase III guidelines and may ultimately lead to a WHO recommendation for LifeNet® LN, it is primarily designed as a randomized controlled trial to compare the relative field durability of three types of LN polymer. Increasing the current average lifespan of LNs is a priority for the WHO. LNs with longer life cycles would need to be replaced less frequently, thus reducing the unit cost of LN distribution and replacement [6, 37]. A WHOPES evaluation criterion based on durability would also create a stimulus for manufacturers to improve performance of their products [6]. Based on current data, WHOPES has been unable to set a fabric durability threshold that a candidate LN should meet. The WHO Technical Expert Group on malaria vector control has proposed the setting of a median LN survival time based on functional LN survival at a given time after distribution. It is hoped that the present comparative study of polypropylene, polyester and polyethylene LNs would provide evidence to help calculate that criterion for different types of LN. Looking forward beyond current pyrethroid LNs to a future of nets treated with alternative insecticides to combat pyrethroid resistant mosquitoes, more durable fabrics may be necessary to maintain LN unit costs to a reasonable level [44, 45].

It is not clear whether the physical durability of LNs in the field can be anticipated by laboratory tests of fabric strength. The WHO consultation that recently reviewed the utility of fabric strength tests concluded that their usefulness for predicting LN durability in the field has yet to be determined, and recommended comparative prospective studies with various types of LN in a single study environment [37]. It is intended that the present study will match the indicators of field durability (hole index and functional survival) with those of laboratory strength tests to predict the durability of LNs in operational settings.

Experience has demonstrated that net attrition can be due to a variety of reasons other than loss of integrity. In the trial of Netprotect® and PermaNet 2.0® LNs in Cambodia, twice as many nets were lost or stolen, sold or given away, than were no longer used due to loss of integrity [43]. In the WHOPES funded evaluation of ICON® Maxx in Tanzania from 2011 to 2014, only 17% of nets survived 3 years of monitoring [46]. While 32% of nets failed due to loss of integrity, 52% were absent for other reasons, including families moving home, seasonal absences, selling or giving nets away. Such losses reduced power to detect an effect from the intervention, and should be discouraged provided the right to withdraw from the trial remains paramount. To encourage responsible net use, study participants/families were requested before recruitment to the cohort component of the trial not to give away or sell the study LNs. They were afforded the freedom to stop using the LNs at any time but to let investigators know the reasons during the next follow up survey. Study participants were informed that LNs would be replaced after 3 years (at the end of the trial period and not before) regardless of net condition but only on production of the trial net. If participants stopped using the LN for any reason, including accumulation of holes, they were to store the net for replacement, or give it to the investigators who would replace it after the trial period has elapsed. These revisions to Phase III guidelines were approved by the WHO and reported by the WHOPES working group in 2014 [46]. Such consent by participants would fulfil the needs of the trial and potentially reduce non-attrition losses, but would not affect participants’ right to stop using their net at any time with impunity.

### Trial status

Monitoring period year 2.

## Abbreviations

AE: Adverse event
AI: Active ingredient
CIPAC: Collaborative International Pesticides Analytical Council
DMO: District medical officer
GC: Gas chromatography
GCP: Good clinical practice
GFATM: Global Fund to Fight AIDS, Tuberculosis and Malaria
GPS: Global positioning system
ID: Identification code
IRS: Indoor residual spraying
LN: Long-lasting insecticidal net
LSHTM: London School of Hygiene and Tropical Medicine
MRCC: Medical research coordinating committee
NIMR: National Institute for Medical Research
NMCP: National Malaria Control Program
PI: Principal Investigator
UCC: Universal coverage campaign
WHO: World Health Organization
WHOPES: WHO Pesticide Evaluation Scheme
